# The risk of coronavirus disease 2019 (COVID-19) among individuals with monoclonal B cell lymphocytosis

**DOI:** 10.1038/s41408-022-00754-x

**Published:** 2022-11-22

**Authors:** Sameer A. Parikh, Sara J. Achenbach, Kari G. Rabe, Aaron D. Norman, Nicholas J. Boddicker, Janet E. Olson, Timothy G. Call, James R. Cerhan, Celine M. Vachon, Neil E. Kay, Esteban Braggio, Curtis A. Hanson, Susan L. Slager, Tait D. Shanafelt

**Affiliations:** 1 Division of Hematology, Mayo Clinic, Rochester, MN USA; 2 Division of Computational Biology, Mayo Clinic, Rochester, MN USA; 3 Division of Clinical Trials and Biostatistics, Mayo Clinic, Rochester, MN USA; 4 Division of Epidemiology, Mayo Clinic, Rochester, MN USA; 5 Department of Hematology and Oncology, Mayo Clinic, Phoenix, AZ USA; 6 Department of Laboratory Medicine and Pathology, Division of Hematopathology, Mayo Clinic, Rochester, MN USA; 7 Department of Medicine, Division of Hematology, Stanford University, Stanford, CA USA

**Keywords:** Chronic lymphocytic leukaemia, Infectious diseases

Severe acute respiratory syndrome coronavirus 2 (SARS-CoV-2), the underlying cause of coronavirus disease 2019 (COVID-19), is a novel coronavirus that has caused a global pandemic, with more than 84 million cases and over 1 million deaths reported in the United States (US). Patients with chronic lymphocytic leukemia (CLL) are at particularly high risk for developing the severe disease when infected by SARS-CoV-2. This increased risk is thought to be secondary to a dysregulated immune system inherently associated with CLL [1], in addition to CLL treatments that can further exacerbate immunosuppression and blunt response to vaccination [2, 3]. In two large retrospective series of CLL patients infected with SARS-CoV-2 [4, 5], the case fatality rate was 35%; patients with previously untreated CLL and those on active treatment had similar outcomes. Monoclonal B cell lymphocytosis (MBL), a precursor condition to CLL, is defined as an absolute clonal B cell count of <5 × 10^9^/L, in the absence of lymphadenopathy, splenomegaly, and cytopenias [6]. MBL is one of the most common pre-malignant conditions in humans, affecting approximately 5–10% of adults over the age of 40 years [7, 8]. MBL can be further subdivided into low-count MBL (typically identified through screening and defined by an absolute B-cell count <0.5 10^9^/L) and high-count MBL with absolute B cell count 0.5–4.9 × 10^9^/L (typically identified in the clinic). Recent data indicate that both low- and high-count MBL are at high risk of developing serious infections requiring hospitalization relative to healthy individuals without MBL [7, 9], suggesting that immune dysfunction in CLL develops early during B cell leukemogenesis. To our knowledge, there are no studies examining the risk of COVID-19 infection in MBL.

In this study, we aimed to describe the risk of contracting SARS-CoV-2 and hospitalization due to COVID-19 infection in MBL. We screened for MBL in a randomly selected subset of individuals in the Mayo Clinic Biobank, a large-scale bio-repository of adult patients assembled to study a wide range of health conditions, as previously described [7, 10, 11]. Although the distinction between high-count MBL and low-count MBL among individuals where a clonal B cell population is present in the peripheral blood is made according to the absolute clonal B cell count; we did not have a peripheral blood complete blood count available for all patients in our cohort. Therefore, we used the previously published and established approach of defining high-count MBL as those individuals where clonal B cells represented ≥85% of total B-cells [12, 13]. For the present analysis, study participants were included if they were residents of Olmsted County, MN, as of March 1, 2020, and had no report of another hematologic malignancy prior to this date. All participants were followed until the earliest of: 1) development of the event of interest (COVID-19 infection or COVID-19 hospitalization); 2) migration from Olmsted County; 3) death; or 4) December 31, 2021. If a subject developed another hematologic malignancy after March 1, 2020, they were censored on the date of the malignancy. To evaluate the natural history of COVID infection (rather than a differential response to vaccine) in MBL, subjects were censored on the date of the first vaccination against COVID-19 (the earliest date of vaccination in this study was December 17, 2020). A diagnosis of COVID-19 infection was ascertained by standard polymerase chain reaction (PCR) testing. COVID-19-related hospitalization was ascertained by reviewing the electronic health record for the ICD-10 diagnosis code U07.1 as a hospital discharge code. The Rochester Epidemiology Project (REP) [14], a population-based medical records-linkage system with access to the complete (in-patient and out-patient) medical records from all medical providers in Olmsted County, was utilized for electronic pulls of COVID-19 testing and for hospitalizations due to COVID-19. Deaths due to COVID-19 infection were identified by reviewing the causes of death listed on the Minnesota death certificate for each deceased subject. The cumulative incidence of COVID-19 infection or COVID-19 hospitalization adjusting for the competing risk of death was also estimated. Cox regression models, adjusted for age, sex, and Charlson co-morbidity index (CCI), were used to compare COVID-19 infection and hospitalization rates and overall survival (OS) between MBL cases and individuals with normal immunophenotype (i.e., without MBL). The Mayo Clinic Institutional Review Board approved this study.

Included in the study were a total of 699 individuals with MBL (660 [94.4%] with low-count MBL and 39 [5.6%] with high-count MBL) and 2916 without MBL. Individuals with MBL were older (median age, 75 years) and more likely to be male (47%) compared to those without MBL (median age, 68 years; 34% male, Table 1). Overall, 147 individuals had a positive COVID-19 PCR test result during a cumulative 3534 person-years of follow up. This included 120 without MBL who tested positive after 2857 person-years of follow-up and 27 with MBL after 638 person-years of follow-up (Fig. 1a). Compared to individuals without MBL, the unadjusted hazard ratio (HR) for risk of contracting SARS-CoV-2 in MBL was 0.96 (95% CI: 0.63–1.45, *P* = 0.83), and similar when adjusting for age and sex [HR = 1.05 (95% CI: 0.68–1.61, *P* = 0.83], and CCI [HR = 0.93 (95% CI: 0.61–1.42, *P* = 0.75)]. The results did not change when participants with MBL were analyzed according to whether they had low-count or high-count MBL, acknowledging low power to evaluate high-count MBL.

**Table 1:** Baseline characteristics of all subjects.

Characteristic	Individuals with normal immunophenotype	Individuals with MBL^a^	Total
N	2916	699	3615
Median Age, years (range)	68 (29–101)	75 (44–98)	70 (29–101)
Male, N (range)	1003 (34%)	330 (47%)	1333 (37%)
Median severity weighted Charlson Co-morbidity index (range)	1 (0–17)	2 (0–19)	1 (0–19)

^a^660 individuals with low count MBL and 39 individuals had high count MBL.

**Fig. 1:**
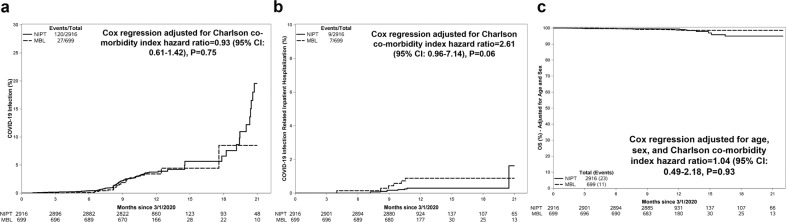
Cumulative incidence of SARS-CoV-2 infection, hospitalization due to COVID-19, and overall survival among individuals with monoclonal B cell lymphocytosis (MBL) compared to those without MBL (i.e., with normal immunophenotype [NIPT]). **a** Cumulative incidence of contracting SARS-CoV-2 adjusted for the competing risk of death in individuals with monoclonal B cell lymphocytosis (MBL) compared to individuals without MBL (normal immunophenotype [NIPT]). **b** Cumulative incidence of COVID-19 infection-related hospitalizations adjusted for the competing risk of death among subjects with monoclonal B cell lymphocytosis (MBL) compared to individuals without MBL (normal immunophenotype [NIPT]). **c** Overall survival of all study participants grouped according to monoclonal B cell lymphocytosis (MBL) or no MBL (normal immunophenotype [NIPT]) at study entry.

During the study, 16 individuals were hospitalized for COVID-19 infection, including 9 individuals without MBL, and 7 individuals with MBL (6 low-count MBL; 1 with high-count MBL, Fig. 1b). Compared to individuals without MBL, the unadjusted HR for risk of COVID-19 infection-related hospitalization was 3.29 (95% CI: 1.23–8.85, *P* = 0.02) among those with MBL. After adjusting for age, the corresponding HR for MBL was 2.55 (95% CI: 0.92–7.04, *P* = 0.07), and after adjusting for CCI, the corresponding HR for MBL was 2.61 (95% CI: 0.96–7.14, *P* = 0.06). Thirty-four individuals died including 23 without MBL and 11 with MBL (Supplemental Table 1). After adjusting for age, sex, and CCI, the HR for death among individuals with MBL was 1.04 (95% CI: 0.49–2.18, *P* = 0.93, Fig. 1c).

Despite the low risk of progression to CLL-requiring therapy, individuals with MBL have a distinct clinical phenotype with important clinical consequences [7, 9]. In a retrospective study from our group, the risk of serious infections (defined as those requiring hospitalization) was significantly increased in high-count MBL compared to age- and sex-matched healthy controls seen in the general medicine clinic [9]. In a population-based cohort study of the general population conducted in Salamanca, Spain, Criado and colleagues screened 639 subjects (median age = 70 years) for MBL using a highly sensitive flow cytometry assay [15]. Infections leading to death were significantly more common in individuals with low-count MBL compared to age- and sex-matched healthy controls without MBL (21% vs. 1.4%, respectively; *P* ≤ 0.001) [15]. Similar results were also recently reported from a community-based cohort of US adults enrolled in the Mayo Clinic Biobank [7].

In the present study of Olmsted County residents, we found that a diagnosis of MBL was *not* associated with a higher risk of contracting SARS-CoV-2 infection but was associated with a 3-fold increased risk of hospitalization due to COVID-19, compared to individuals with no MBL. These findings were independent of age and co-morbidity status, suggesting that the presence of MBL per se is associated with increased risk for severe COVID-19 infection, although the effect attenuated when adjusting for other factors. The biologic underpinnings of an increased risk of serious infection in individuals with MBL are not completely understood. Although the rates of hypogammaglobulinemia are not significantly different between MBL and those without MBL [9], preliminary data indicate that individuals with high-count MBL may have significant T cell dysfunction, including a higher number of exhausted T cells and a defective T cell immunological synapse [16], as observed in CLL. Whether these or other aspects of immune dysfunction contribute to our findings will require a comprehensive evaluation of the immune system in MBL.

Our study has several strengths. It is one of the first studies to examine the risk of contracting SARS-CoV-2 infection and developing severe COVID-19 requiring hospitalization in a large cohort of individuals with MBL in the United States. All study participants were followed systematically, with near complete data on reasons for death available for all. However, our study also has several limitations. Although we had access to all medical records from Olmsted County residents, ascertainment bias for hospitalization with COVID-19 infection outside the county is possible. Most study participants were White; hence, these results may not be generalizable. The findings reflecting the natural history of SARS-CoV-2 infection before broad-scale vaccination was approved is both a strength and a limitation of our investigation. Finally, despite including >3500 participants in this study, the total number of hospitalization and infection events was low, which limited our ability to adjust for other confounders and evaluate subgroups.

In summary, in this study evaluating the risk and severity of COVID-19 infection among Olmsted County residents with and without MBL prior to the introduction of SARS-CoV-2 vaccinations, we found that the risk of acquiring SARS-CoV-2 infection was not different among individuals with or without MBL. However, once infected with COVID-19, those with MBL have a higher risk of hospitalization due to COVID-19, suggesting that immune dysregulation increases the risk of adverse outcomes. These findings add to the mounting evidence that MBL is associated with clinically significant impairment of immune function. Given that MBL is a largely unstudied condition affecting over 5-10% of adults over the age of 40 and the increasing incidence with aging, these findings have potentially large population health implications.

## Supplementary information

Supplemental Table 1

## Data Availability

Data generated and/or analyzed during the current study are available from the corresponding author on reasonable request.
