# Supplementary material for: The risk of coronavirus disease 2019 (COVID-19) among individuals with monoclonal B cell lymphocytosis
Source: Blood Cancer J. 2022 Nov 22;12(11):159. doi: 10.1038/s41408-022-00754-x (PMC9684458; doi:10.1038/s41408-022-00754-x)
Supplement: Supplementary file 1 — Supplemental Table 1 [file 41408_2022_754_MOESM1_ESM.docx]

**Supplemental Table 1: Causes of death (n=34) among the study subjects who died during follow-up**

| **Cause of Death** | | **N** |
| --- | --- | --- |
| Infection (not COVID-19) | | 2 |
| Malignancy | Lung | 4 |
|  | GI tract, including pancreas | 3 |
|  | Renal | 1 |
|  | Unknown primary | 3 |
| Chronic obstructive lung disease | | 3 |
| Alzheimer’s disease | | 3 |
| Ischemic heart disease | | 6 |
| Cerebrovascular disease | | 4 |
| Other* | | 4 |
| Death certificate unavailable | | 1 |
| **Total** | | **34** |

*other includes obesity related complications, fall, respiratory arrest, and inhalational lung injury
